# The effect of self-disclosure on mass trust through TikTok: An empirical study of short video streaming application users

**DOI:** 10.3389/fpsyg.2022.968558

**Published:** 2022-08-18

**Authors:** Athapol Ruangkanjanases, Ornlatcha Sivarak, Din Jong, Yajun Zhou

**Affiliations:** ^1^Chulalongkorn Business School, Chulalongkorn University, Bangkok, Thailand; ^2^Mahidol University International College, Mahidol University, Nakhon Pathom, Thailand; ^3^Digital Design and Information Management, Chung Hwa University of Medical Technology, Tainan, Taiwan; ^4^College of Finance and Economics, Nanchang Institute of Technology, Nanchang, China

**Keywords:** self-disclosure, mass trust, perceived similarity, trust disposition, short video application, social media

## Abstract

In the short video application scenario, self-disclosure helps to establish and maintain relationships with others, and is important for the formation of mass trust. To this end, this study investigates the impact of self-disclosure on mass trust in the context of short video applications based on social exchange theory, and introduces perceived similarity to explain the specific impact mechanism while focusing on the boundary conditions of trust disposition in it. This study takes TikTok as the research object and analyzes the data obtained from the questionnaire survey. The empirical test results show that self-disclosure not only affects mass trust directly, but also indirectly through perceived similarity. In addition, a moderating effect of trust disposition on perceived similarity was found to affect mass trust. The findings of this paper contribute to an in-depth understanding of the potential intermediate mechanisms and boundary conditions of self-disclosure on mass trust, reveal the theoretical relationship between self-disclosure and mass trust, bridge the gap between previous mechanisms of mass trust influence from the perspective of empirical research, and effectively guide the management practice of short video applications.

## Introduction

In recent years, short video applications have become one of the fastest growing mobile Internet segments in terms of daily active user size, and are growing and receiving continuous attention from academia and industry (Thomas et al., 2019; Zhang, 2021). Studies have shown that short video applications have penetrated various industries and influenced all aspects of the public's life (Sheth and Kellstadt, 2021), enabling easy information dissemination, communication and sharing among users (Zhang et al., 2019). Especially with the development of Internet and information technology, short video applications have attracted a large number of active users and gradually become a common tool for public life and socialization (Gao et al., 2020). In this context, short video applications have become the primary tool used by many users to build, maintain and manage relationships with others. Today, short video applications such as TikTok and quick worker are widely available and have become a part of many users' public disclosure of their daily lives. In order to maintain good social relationships, users of short video applications are disclosing various information such as profiles, posts, comments and photos of themselves online (Dwivedi et al., 2021). Short video applications are designed to build and maintain relationships with people by disclosing information about themselves and have the positive aspect of forming social capital (Chen, 2018; Tifferet, 2019; Pu et al., 2021). There is no doubt that in an increasingly competitive market, self-disclosure has become a key issue for short video apps to think about to attract and retain users (Walker et al., 2021).

Self-disclosure is when people present information about themselves to others, such as personal status, tendencies, past events, and future plans (Choi and Bazarova, 2015; Zhang and Fu, 2020). Therefore, self-disclosure often occurs during communication between people, and all disclosed personal information is public (Tsay-Vogel et al., 2018). In general, such self-disclosure is a prerequisite for the formation of social relationships (Aboulnasr et al., 2021). Nowadays, short video applications have become the main channel of online communication among the user. In the context of short video applications, users not only engage in positive communication activities (self-disclosure), but also have a greater need to gain the trust of the public. Self-disclosure usually takes into account the outcomes from risks and benefits (Arpaci, 2020). However, due to the low barriers to entry in the short-video application market, it is often difficult for individuals' rights and interests in the short-video application scenario to be adequately protected, making individuals worry that public disclosure of information will lead to related negative issues, which exacerbates the gradual decrease in public trust in short-video applications (Wang and Liu, 2019).

Trust becomes more important due to the higher uncertainty faced by the user due to the lack of face-to-face interaction with offline and comments from other users. In the context of short-form video applications, self-disclosure helps to mitigate individual perceived uncertainty and risk, plays a positive counteracting role to the risk of privacy leakage (Hofstetter et al., 2017), and is an important factor in establishing and developing trust among the user. However, fewer studies have explored and examined the mechanisms and boundary conditions underlying the effect of self-disclosure on mass trust in the context of short-form video applications. Given the important role of self-disclosure in the development of short video applications and mass socialization activities (Thompson and Brindley, 2021), this study investigates the potential intermediate mechanisms by which self-disclosure affects mass trust from the perspective of social exchange theory. It also provides relevant empirical research support based on existing relevant studies.

According to the social exchange theory (Blau, 1964; Lu et al., 2010; Shen et al., 2010; Fu et al., 2018; Wang et al., 2020), perceived similarity is a potential intermediate mechanism for the effect of self-disclosure on mass trust in the context of short video applications, which helps to explain the role of self-disclosure in influencing the relational outcomes of mass trust, usage behavior, and creativity. Under the influence of self-disclosure, individuals perceive high quality perceived similarity relationships with others, and perceived similarity tends to inspire personal identity, shared characteristics, and trust (Fu et al., 2018) and motivates individuals to reciprocate by responding to higher trust and good relationships through short video applications (Lu et al., 2010). For example, like, comment, retweet and display your own work online. Individuals actively express their feelings and thoughts and release repressed emotions through short video applications, which in turn make them an important channel for influencing their social activities, and as the degree of this influence increases, so does the self-disclosure, thus increasing the trust of others. Moreover, the enhancement of trust is an important factor to promote the development of short video applications. However, whether and how self-disclosure affects public trust in the context of short video applications needs to be verified through empirical studies. Therefore, in order to explore the intermediate mechanisms through which self-disclosure affects public trust, this paper will examine how self-disclosure affects public trust through the potential mechanism of perceived similarity. In this paper, we will explore this issue in two separate paths: the direct influence role of self-disclosure on the trust of large subordinates and the indirect influence role through perceived similarity.

Although self-disclosure can influence individuals' trust, existing research “does not know much about whether individuals with different personal characteristics respond differently to perceived similarity” (Zeng, 2017). Trust disposition is an important measure of an individual's personality traits and dispositional tendencies to trust others (Cheng et al., 2016). Trust disposition is intended to reflect the characteristics that individuals have in themselves, not their trust intention or attitude toward a specific object. Such individual characteristics are mainly related to the individual's cultural background, personality type, and past experiences, and therefore trust disposition varies greatly among different types of individuals (Lu and Zhou, 2007). Because trust disposition reflects an individual's disposition, it usually needs to be combined with factors that individuals perceive as reliable in order to jointly form trust in others, so trust disposition is often used as a moderating variable to influence the relationship between trustworthiness factors and trust (Gefen, 2000). Mo and Luo (2019) suggest that trust disposition may influence the role of perceived similarity on individual trust. Trust disposition is not only one of the cultural values that receive the most attention in virtual contexts such as short video applications (Grabner-Kräuter, 2009), but is also the moderating variable most closely related to the study of social interactions (Jiang, 2017; Shi et al., 2021). Therefore, based on existing studies (Zhou and Xu, 2021), this study further investigated the role of individual differences in personal cultural values moderating the effect of self-disclosure on trust, i.e., it further examines the effect of culturally specific personal trust disposition on the relationship between perceived similarity and mass trust moderating role.

## Theoretical foundation and hypothesis development

### Social exchange theory

Homans (1958) proposed the core view of social exchange theory: all human behavior was exchange behavior, including material and immaterial exchange; human beings participate in exchange behavior to satisfy their own interests, and rationally calculate their own payoffs and rewards in the exchange, trying to achieve fair exchange. Social exchange theory suggested that individuals need to reciprocate the value they have received in social interactions in order to maintain the benefits of the relationship, i.e., to maintain a positive relationship by performing behaviors that benefit the other person, which was a “reciprocal need” that drives the social exchange behavior (Yu, 2004). The values and benefits obtained from the interaction directly drive individuals to form identification, attachment, and willingness to maintain the relationship with the other party, and in order to continue to benefit from the interaction, individuals were motivated to construct and maintain the relationship by performing behaviors that benefit the other party, which essentially belong to the category of social exchange (Gouldner, 1960). Social exchange theory was a theory that studies social interactions and exchange relationships between individuals or organizations, which was based on two important assumptions. Namely, the assumption of tendency to avoid harm in terms of human nature and the assumption of reciprocal dependence in terms of interpersonal relationships (Blau, 2017). The core logic was that human exchange behavior was based on rewards and costs, and individuals were motivated to exchange if the exchange can produce was positive and positive outcomes. Social exchange includes formal exchange mechanisms about money, goods, and services based on contractual behaviors, and informal exchange mechanisms based on non-contractual behaviors such as gratitude, pleasure, approval, appreciation, love, and stimulation (Zhang and Xuna, 2016).

Based on the social exchange perspective, individual self-disclosure on short videos attracts others to interact, and in the process of interaction, both parties communicate, and others can perceive their own exchange value from the process of communication. In the context of short video applications, as a formal exchange mechanism aspect and important research variable was self-disclosure. Self-disclosure refers to the process by which individuals reveal their thoughts, feelings, and experiences to others. Simply put, self-disclosure was the act of individuals actively disclosing personal information to others (Tsai and Ghoshal, 1998); the variable studied as an informal exchange mechanism was trust. Trust was a social exchange behavior carried out in self-disclosure and trust of others, and the characteristics of the relationship between the two parties are the condition and basis of social exchange, and the formal and informal exchange mechanisms complement each other to ensure the smooth process of social exchange; and perceived similarity was regarded as an important variable representing the characteristics of both parties of exchange. In the context of self-disclosure, individuals can infer their similarity to the discloser from the self-disclosure information, generate interpersonal attraction, hold high identification and positive evaluation of the discloser, and thus form trust. From a social exchange perspective, when individuals engage in self-disclosure, they expect others to self-disclose as well, exchanging information or initiating interactions with each other, a process that increased mutual intimacy (Park et al., 2011). Therefore, this study explores how self-disclosure affects perceived similarity and thus mass trust based on short video application contexts from social exchange theory.

### Self-disclosure and mass trust

Self-disclosure refers to an individual's ability to reveal previously unknown information to the public and make it shared knowledge, with the goal of creating good emotional bonds with the public and increasing public trust in the self (Joinson and Paine, 2007; Towner et al., 2022). Self-disclosure contributes to the development of intimacy, and as intimacy develops, the depth and breadth of self-disclosure increases (Gentina and Chen, 2018). This, in turn, further promotes an emotional response to the intimate relationship between the masses and the self (Hollenbaugh, 2011; Wang and Stefanone, 2013; Chen et al., 2018). Research shows that the self-disclosure of online information varies depending on the context, and the online context makes the self-disclosure significantly higher than the offline condition due to the interaction, communication and exchange between users (Jiang et al., 2011; Fox et al., 2021). In this sense, individual self-disclosure strategies may play a crucial role in the expected interpersonal relationships in the short video application scenario, where the user tends to be placed in an environment of decreasing information uncertainty, which in turn motivates information publishers to keep disclosing information (Gibbs et al., 2011; Christofides et al., 2012; Tokas, 2022).

In the short video application scenario, mass trust is a central issue affecting online interpersonal interactions and plays an important role in intimate relationships. Existing research suggested that in intimate relationships, mass trust was hardly constructed spontaneously by individuals, but was based on third-party assurance and advice, and that mass trust cannot be enhanced without a stable and reliable third party (Lane and Bachmann, 1996). Based on this, in the short video application scenario, mass trust can be established through the mechanism based on the short video application platform, for example, according to the constraints of the platform, individuals have to self-disclosure of information (Grov et al., 2013; Zhong et al., 2021).

The study shows that self-disclosure as proactive disclosure of relevant information to users mainly includes basic personal information, interests, values and other voluntary disclosures (Chen, 2013). Self-disclosure by individuals on a virtual platform can bring interpersonal trust, commitment, and intimacy closer and facilitate relationship development among individuals (Park et al., 2011; Ramos et al., 2019). On the one hand, users realize self-disclosure through the process of gradually presenting personal information on short video applications, and the process of information disclosure can promote the masses' dynamic understanding of self, thus reducing the uncertainty of the masses' perception of self and mitigating the masses' perceived risk, thus increasing mass trust (Gibbs et al., 2006; Wang and Stefanone, 2013; Li et al., 2018). On the other hand, self-disclosure is a form of social exchange, and self-disclosure will increase the possibility of interaction between the public and the self, which will enhance the public's sense of familiarity and intimacy with the self and increase trust in intimate relationships (Deng et al., 2021). Clearly, self-disclosure helps to enhance others' trust in the self. Based on the aforementioned arguments, we hypothesize the following.

H1: Self-disclosure is positively related to mass trust.

### Self-disclosure and perceived similarity

Perceived similarity is an important antecedent influencing factor of interpersonal attraction (Solomonov and Barber, 2018). Wang and Stefanone (2013) considered perceived similarity as the degree of similarity between perceived others and themselves in terms of values, interests, attitudes, preferences, etc. Therefore, self-disclosure on short video applications may be considered as a general pattern of behavior in the context of large audiences. With the increase in individual self-disclosure information and the construction of user identities (Pechmann et al., 2021), individuals with a strong desire to be identified with the public tend to use strategies that are perceived as positive and worthy of replication to demonstrate desirable characteristics and behaviors, thereby attracting more disclosure (Rosenberg and Egbert, 2011; Wei and Liu, 2020).

Like previous social media, short-form video applications are by their nature virtual platforms built around users' interpersonal relationships, and thus are not anonymous environments. Instead, short video applications provide users with an open platform through which they can generate content and selectively self-disclosure to build and manage their image and identity (Walther et al., 2009). Self-disclosure not only helps users present themselves from multiple perspectives, such as values, interests, attitudes, preferences, etc., but also the more and more information self-disclosure presents, the more it helps others infer individuals with similar characteristics to themselves from publicly disclosed information, thus increasing perceived similarity (Hooi and Cho, 2014).

One of the key features of short-form video applications is user-generated content. Researchers have found that short video applications, such as TikTok, allow users to display self-disclosure in a way that they are very aware of their self-presentation strategies (Rosenberg and Egbert, 2011; Solomonov and Barber, 2018) and create identities. In more detail, individuals with higher self-disclosure characteristics are more likely to engage in self-promotional content or behavior on short videos and better present themselves on large social networks (Mehdizadeh, 2010; Ong et al., 2011; Carpenter, 2012; Malloch and Zhang, 2019), thus attracting friends who share their interests. To elaborate, TikTok self-disclosure creates opportunities for users to selectively present themselves with highly self-controlled content so that others can form perceived similarity based on the selective disclosure. Self-disclosure is the act of making oneself known to the public; it was the process by which individuals actively reveal personal information, and this process can trigger perceived similarity among the public (Solomonov and Barber, 2019). Good interaction between users on short video applications positively affects perceived similarity, which in turn generates interpersonal attraction and raises relationship levels (Pechmann et al., 2021). In particular, when the self-disclosure information is more adequate, it will lead to more people perceiving stronger similarity and thus higher perceived similarity to the individual who publicly discloses the information. Self-disclosure is an important antecedent variable affecting perceived similarity (Tran et al., 2022). As such, we expect that brand identity will drive consumers to attain higher purchase intention.

H2: Self-disclosure is positively related to perceived similarity.

### Perceived similarity and mass trust

Perceived similarity facilitates the establishment of trust mechanisms and has a positive impact on the establishment and development of mass trust. Perceived similarity is a necessary prerequisite for the establishment of mass trust, and trust is the response of individuals who feel similar to the discloser in terms of characteristics such as vision, values and rules of behavior (Lu et al., 2010; Crijns et al., 2017). From a social psychological perspective, relationship satisfaction was influenced by perceived similarity in a relational context (Pearce et al., 2019), which supports the positive impact of perceived similarity on trusting behavior from the evidence. The literature on marketing suggests that individuals' behaviors, goals, and policies reflect shared interests and value orientations between the interacting parties and are important antecedents for assessing the influence of perceived similarity on audience trust in self. Mass trust in intimate relationships was significantly influenced by perceived similarity based on shared values and beliefs (Dwyer et al., 1987; Morgan and Hunt, 1997; Siegrist and Hartmann, 2020).

The same trust mechanisms exist for intimate relationships in virtual contexts. For example, in a book recommendation system, individuals tend to adopt recommendations that are more similar to their values and beliefs (Ziegler and Golbeck, 2007; Zeng et al., 2019). When people gather in the same short video application platform, they tend to view others on that platform in a positive way, which apparently enhances their trust (McKnight et al., 1998; Casalo et al., 2013). Perceived similarity may involve several aspects such as demographic characteristics, interests, and values, so mass trust can be formed through similarity of individuals in terms of characteristics, interests, and values. The general masses are prone to identify with individuals whose values are similar to theirs, and this identification usually leads to trust in the identified individual (Boyd et al., 2019). As the degree of similarity between individuals' perceptions and others increases, the level of mass trust in self will gradually increase.

Therefore, the positive effect of high-perceived similarity between individuals with mass trust was enhanced in the short video application context with trust disposition, quality-assured shared information, familiarity, and other third-party endorsements. Given that individuals and the user have similar value characteristics, the user can form perceived risks to individuals based on common goals or interests, thus enhancing their trust in individuals; when the user relates to individuals with more consistent similarity, they can achieve better communication and understanding, reduce disagreements and conflicts, enhance mutual intimacy, and thus increase trust. Taken together, perceived similarity has a positive effect on mass trust.

H3: Perceived similarity is positively related to mass trust.

### Mediating role of perceived similarity

Currently, the concept of perceived similarity is widely studied in the fields of sociology, psychology and marketing. Perceived similarity is an important measure to assess whether users on social applications share common characteristics among themselves, including demographic characteristics such as individual interests, hobbies, values, and consumption levels (Lu et al., 2010; Liu et al., 2017; Zhan and Du, 2018; Yan and Fu, 2021). Perceived similarity motivates individuals to strongly prefer relationships with people who have similar characteristics to themselves (Lu et al., 2010). Thus, the hypothesis that perceived similarity can lead to attraction between individuals has been proposed and tested, and individuals also prefer those with similar attitudes (Fisher, 1974) or interests (Martin et al., 2013). In addition, Guéguen et al. (2011) also demonstrated that perceived similarity has a facilitating effect on people's implicit behavior. Lu et al. (2010), using a virtual community setting as a research context, further confirmed that perceived similarity plays a significant influence on the construction of trust processes among online users in a study to verify whether perceived similarity has a positive relationship on trust of other users in the community, and also found that the effect of self-disclosure on trust is mediated by perceived similarity.

According to the social response theory proposed by Reeves and Nass (1996), people will apply realistic social norms to computers with human characteristics. Thus, in short video application contexts, perceived similarity can reduce reactions by increasing compliance and reducing resistance as well as facilitating individuals to accept the information provided by the communicator, stimulating connections with other members of the community, and can increase trust relationships. This type of perceived similarity can be based on the individual's self-disclosure (So, 2018). For example, individuals present demographic information about themselves to the user through short video applications (Hitsch et al., 2010; He et al., 2016), as well as aspects of their thoughts, feelings, and experiences. Social psychological research on interpersonal attraction has shown that self-disclosure is an important means for individuals to develop online relationships. With self-disclosure, individuals find friends who share similar characteristics with them and achieve trust building (Pearce et al., 2019). When individuals search for and interact with friends with similar values to their own through the public information of self-disclosure, they will experience smoother communication and better understanding, and they will be able to predict each other's reactions and thus be more likely to elicit trust from others. Comprehensive analysis of the above, we thus hypothesize:

H4: Self-disclosure and mass trust are mediated by perceived similarity.

### Moderating role of trust disposition

Trust disposition is the tendency to assess the extent to which the individuals trusts others (Cheng et al., 2016). Trust disposition is not disturbed by the external environment but is associated with personal characteristics (Cheng et al., 2016), and it works on the premise that others are usually well-meaning, reliable, and ethical. Studies have argued that trust disposition appears to be more important in situations where the context and type of relationship is unknown (Shen et al., 2010; Fu et al., 2018). The relative lack of “media richness” in the short-form video application scenario presents many uncertain risks and hidden concerns that could adversely affect the enhancement of mass trust (Daft et al., 1987; Cheng et al., 2016). Although the popularity of intimate relationships on virtual platforms such as Facebook, MySpace, and Bebo has grown dramatically, supported by information technology, and in opposition to previous theories about people's ability to trust online. However, at a practical level, individuals are more willing to interact through short video application platforms and to post comments and share personal opinions, suggesting to some extent that the public's level of trust concerns about virtual platforms has eased or, indeed, that they have less control over tools and technologies that interfere with trust. An important feature of the short video application scenario is trust disposition, which has been confirmed in previous studies that trust disposition responds positively to the development of mass trust and the deepening of self-disclosure (Castelfranchi and Falcone, 1998).

Research has shown that in virtual contexts, although users can graft emotional ties with individuals through technology, technology itself does not have an emotional function, making it difficult to provide intimate connections to the public because the balance between building deep trust lies in social factors and the “natural language” connection between users. Therefore, in the context of short video applications, the establishment of mass trust also needs to be moderated by social factors and the “natural language” between users (Kolb et al., 2008), and the propensity to trust is an important indicator of social factors and the “natural language” between users. It is an important indicator of the “natural language” between social factors and users. It reflects human-computer (or system) trust, interpersonal trust relationships and personality trust, as well as risks and attitudes and potential benefits (Cheng et al., 2016). In the short video application context, when the individual's trust disposition is higher, his perceived similarity is also stronger, and when the individual's perceived similarity keeps increasing, the mass trust in him also increases. In other words, when the individual's trust disposition keeps increasing, the perceived similarity also increases, and the establishment and development of mass trust also increases. Clearly, as trust disposition increases, the positive effect of perceived similarity on mass trust follows. Conversely, as trust disposition decreases, the positive effect of perceived similarity on mass trust also decreases. It can be inferred that in the short video application context, the individual's trust disposition has a moderating effect on perceived similarity and mass trust. Comprehensive analysis of the above, we thus hypothesize:

H5: Perceived similarity and mass trust are moderated by trust disposition.

**Figure 1:**
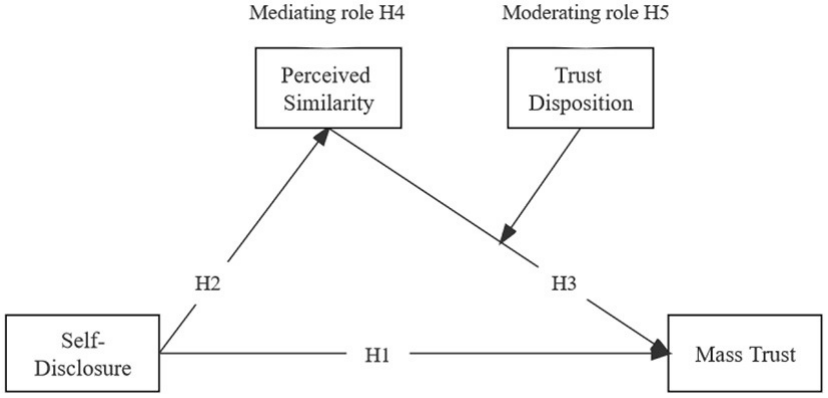
Theoretical model.

In summary, this study proposes a research model as shown in Figure 1.

## Methods

### Participants and procedure

In this study, TikTok was chosen as the research sample. The reason for choosing TikTok as the research object is that since its launch on September 20, 2016, TikTok has been a short video community platform for all ages, through which users can upload short videos to share and record their good lives. Currently, TikTok has become the most influential and representative short video application in China. This study adopted a questionnaire survey to collect data and mainly adopted an online platform to distribute questionnaires. The method was to generate a web-based questionnaire with the help of the web platform provided by “Questionnaire Star”, and then publish the link to the questionnaire through WECHAT. In order to improve the efficiency of the questionnaire collection, a reward of six RMB was given to each user who fills in the questionnaire effectively. The questionnaire survey was started on November 9, 2021 and ended on January 16, 2022. A total of 432 questionnaires were distributed through this method, and 395 valid questionnaires were obtained after eliminating invalid questionnaires, with a sample validity rate of 91.4%. The details are shown in Table 1.

**Table 1:** Descriptive statistical analysis.

**Variables**	**Item**	**Frequency**	**%**
Gender	Male	128	32.4
	Female	267	67.6
Age (year)	19 or less	47	11.9
	20–29	288	72.9
	30–39	18	4.6
	40–49	22	5.6
	50 or above	20	5.1
Marriage	Married	58	14.7
	Unmarried	334	84.6
	Divorce	3	.8
Occupation	Student	270	68.4
	Freelance	17	4.3
	Executive in private enterprise	17	4.3
	Civil servant	33	8.4
	Clerk in state owned enterprise	20	5.1
	Executive in private enterprise	38	9.6
Education	College and blow	22	5.6
	Undergraduate	52	13.2
	Master's degree and above	321	81.3
Consumption (RMB)	Below 2,00	160	40.5
	2,000–3,999	104	26.3
	4,000–5,999	57	14.4
	6,000 or more	74	18.7
Continuous use time (year)	Within 6 months	96	24.3
	6 months to 1	69	17.5
	1–2	136	34.4
	Over 3	94	23.8

### Measures

To ensure good reliability and validity of the variables in the study, all the scales in this study were borrowed from the more established scales in previous studies. The scale measures and literature sources are shown in Table 2.

**Table 2:** Variables and measurement item.

**Construct**	**Item content**	**References**
Self-disclosure	SD1. I often talk about my feelings on TikTok	Hollenbaugh, 2011; Wang and Stefanone, 2013
	SD2. I often post something about my relationships and private life on TikTok.	
	SD3. I often post photos of me and my friends on TikTok.	
	SD4. I often express my thoughts and true self completely on TikTok.	
Mass trust	MT1. Individuals in TikTok are in general reliable.	Pavlou and Gefen, 2004; Gibreel et al., 2018
	MT2. Individuals in TikTok are in general honest.	
	MT3. Individuals in TikTok are in general trustworthy.	
Perceived similarity	PS1. I share similar goals with some members on TikTok.	Lu et al., 2010
	PS2. I share similar interests with some members on TikTok.	
	PS3. I share similar aesthetic tastes with some members on TikTok.	
	PS4. I share similar shopping experiences with some members on TikTok.	
Trust disposition	TD1. I generally trust other people.	Cheng et al., 2019
	TD2. I feel that people are generally well meaning.	
	TD3. I feel that people are generally trustworthy.	
	TD4. I feel that people are generally reliable.	

## Data analysis and results

### Outer model evaluation

This study analyzes the outer model according to the views of previous related scholars (Anderson and Gerbing, 1998; Hair et al., 2017). Specifically, if standardized factor Loadings > 0.70 for each scale, Cronbach's Alpha > 0.60, rho_A > 0.60, Composite Reliability (CR) > 0.60, and Average Variance Extracted (AVE) > 0.50 for each factor, it indicates that the outer model of this research owns convergent validity. The results of outer model are shown in Table 3. The range of factor loadings are between 0.726 and 0.924, Cronbach's Alpha is between 0.773 and 0.878, rho_A is between 0.788 and 0.881, CR is between 0.854 and 0.919, and AVE is between 0.594 and 0.790. Thus, the results of standardized factor Loadings, Cronbach's Alpha, rho_A, CR and AVE meet the CFA criteria. Therefore, this indicates that this study has the acceptable convergent validity.

**Table 3:** Outer model results.

**Variables**	**Items**	**Factor loadings**	**Cronbach's alpha**	**rho_A**	**CR**	**AVE**
Self-disclosure (SD)	SD1	0.821	0.773	0.788	0.854	0.594
	SD2	0.760				
	SD3	0.772				
	SD4	0.726				
Mass trust (MT)	MT1	0.869	0.867	0.868	0.919	0.790
	MT2	0.891				
	MT3	0.906				
Perceived similarity (PS)	PS1	0.880	0.878	0.881	0.917	0.734
	PS2	0.906				
	PS3	0.820				
	PS4	0.818				
Trust disposition (TD)	TD1	0.832	0.877	0.881	0.916	0.732
	TD2	0.816				
	TD3	0.924				
	TD4	0.846				

*SD, Self-Disclosure; MS, Mass Trust; PS, Perceived Similarity; TD, Trust Disposition*.

Heterotrait-Monotrait (HTMT) ratio of correlations was used to evaluate the discriminant validity. HTMT should be significantly smaller than 0.90 in order to evidently distinguish between two constrcuts (Henseler et al., 2015, 2016). Table 4 reports the discriminant validity for the outer model. As shown in Table 4, HTMT ratios for each pair are smaller than 0.9; the results indicate that all constructs are explicitly distinguished of each other. Therefore, discriminant validity is satisfied in this research.

**Table 4:** Results of discriminant validity by HTMT.

	**Mass trust**	**Perceived similarity**	**Self-disclosure**
Perceived similarity	0.859		
Self-disclosure	0.746	0.709	
Trust disposition	0.850	0.783	0.802

*SD, Self-Disclosure; MS, Mass Trust; PS, Perceived Similarity; TD, Trust Disposition*.

### Inner model results

We examined the proposed research hypotheses with the results of inner model. The details of the hypothesis testing are shown in Table 5. Self-disclosure (*t*-value = 2.110, *p*-value < 0.05) is positively related to mass trust. Therefore, H1 is significant and supported. That is, self-disclosure has a significant and positive effect on mass trust. Self-disclosure (*t*-value = 11.584, *p*-value < 0.001) is positively related to perceived similarity. Therefore, H2 is verified and supported. That is, self-disclosure has a significant and positive effect on perceived similarity. Perceived similarity (*t*-value = 5.997, *p*-value < 0.001) is positively related to mass trust. Therefore, H3 is supported. That is, perceived similarity has a significant and positive effect on mass trust.

**Table 5:** Regression coefficient.

**Path**	**Path coefficient**	**Standard deviation**	***t*-value**	***p*-value**
H1: SD → MT	0.099	0.047	2.110	0.035
H2: SD → PS	0.597	0.052	11.584	0.000
H3: PS → MT	0.456	0.076	5.997	0.000

*SD, Self-Disclosure; MT, Mass Trust; PS, Perceived Similarity*.

According to Hayes (2009), this study used bootstrap estimation technique to calculate the mediating effect. That is, if zero is not included between lower bound and upper bound of bias-corrected 95% confidence and both *t*-value > 1.96 and *p*-value < 0.05 are satisfied, the mediation effect is indicated to be significant. In this study, the results of the mediating effects analysis were as shown in Table 6. The total effect of self-disclosure on mass trust is 0.372. At the 95% confidence level, zero does not include the bias-corrected 95% confidence interval range (*t*-value = 5.978; *p*-value < 0.001). Therefore, there is a total effect exists. The indirect effect of self-disclosure on mass trust is 0.272, zero does not include the bias-corrected 95% confidence interval range (*t*-value = 4.167; *p*-value < 0.001). Therefore, the indirect effect exists in the proposed model. Then, the direct effect of self-disclosure on mass trust is 0.099, zero does not include the bias-corrected 95% confidence interval range (*t*-value = 2.037; *p*-value < 0.01). Therefore, there is a direct effect in the proposed model. Thus, above results of the study show that H4 is significant and perceived similarity has the partial mediation.

**Table 6:** Mediation effect.

**Path**	**Effect**	**Standard deviation**	***t*-value**	**Bias corrected 95% lower bound**	**Bias corrected 95% upper bound**
Total effect: SD → MT	0.372***	0.062	5.978	0.260	0.502
Indirect effect: SD → PS → MT	0.272***	0.065	4.167	0.175	0.429
Direct effect: SD → MT	0.099**	0.049	2.037	0.013	0.190

***p-value < 0.001.

**p-value < 0.05.

*SD, Self-Disclosure; MT, Mass Trust; PS, Perceived Similarity*.

The moderating effects are reported in Table 7. In the present study, trust disposition (TD) is the moderating variable. The results of structural equation modeling have been shown that the moderator effect of perceived similarity (PS) × trust disposition (TD) on self-disclosure (SD) is 0.067 (*T* Statistics = |2.380| > 1.96, *p*-value < 0.05), implying the presence of a positive moderating effect of trust disposition (TD) on the relationship between perceived similarity (PS) and self-disclosure (SD). Specifically, the slope of perceived similarity (PS) on self-disclosure (SD) increases positively by 0.067 units for each 1-unit increase in the moderating variable trust disposition (TD). That is, trust disposition (TD) has a positive moderating effect. Therefore, H5 is significant.

**Table 7:** The analysis of moderating effect.

**Dependent variable**	**Independent variable**	**Path coefficient**	**Standard deviation**	***T* Statistics**
Mass trust (MT)	Perceived similarity (PS)	0.456***	0.076	5.997
	Trust disposition (TD)	0.381***	0.078	4.857
	Perceived similarity (PS) × Trust disposition (TD)	0.067*	0.028	2.380

*p < 0.05.

***p < 0.001.

## Research and discussion

### Conclusion

First, the results of data analysis indicate that self-disclosure has a significant effect on mass trust. The findings are consistent with the results of Gibbs et al. (2006), Wang and Stefanone (2013), Li et al. (2018), and Deng et al. (2021). It is speculated that the reason for this may be that self-disclosure usually brings psychological utility to people. From the perspective of social exchange, when individuals engage in self-disclosure, they expect others to self-disclose as well, exchanging information or interacting with each other, and this process increases the intimacy between each other. In terms of psychological utility, self-disclosure develops interpersonal relationships, from which people can gain social support, enhance their own wellbeing, and promote the deeper development of trust. In the short video application context, individuals should easily pay attention to each other's feelings about themselves when self-disclosure, so that they can assess their own motivation before self-disclosure (venting their emotions or wanting to enhance their feelings with each other, etc.), as well as their emotional changes after self-disclosure, and feel whether the other party has established an emotional connection and empathy with themselves or has been infected by their own emotions. In other words, self-disclosure with breadth and depth can increase others' good feelings, trust and satisfaction with intimacy. When the ego wants to develop further relationships with others, it can use the convenience of short-view applications to share some personal daily life, or the ego's attitude and feelings about things.

Second, the results of data analysis verified the significant effect of self-disclosure on perceived similarity. The results of the study are consistent with those of Mehdizadeh (2010), Ong et al. (2011), and Carpenter (2012). Self-disclosure can stimulate stimulation of positive emotions in others, and people tend to seek out stimuli with positive experiences in order to experience positive emotions. Moreover, people associate self-disclosure, positive emotions with specific others and develop positive attitudes toward others. In other words, once people experience positive emotions in interactions with others, they will hold a positive attitude toward others, i.e., they are attracted to others. In short video application contexts, the main factors that generate interpersonal attraction are similarity, reciprocity, and agreeable characteristics. In addition, interpersonal attraction can have an impact on people's perceptions and behaviors. On the one hand, the attracted person tends to align with the attractor and is therefore more receptive to the attractor's behaviors, perspectives, and attitudes, resulting in higher identification and more positive evaluations of the attractor. On the other hand, the attracted person also voluntarily increases the frequency, breadth, and intensity of communication with the attractor in order to obtain rewarding stimuli and experience positive emotions from the process.

Third, the results of data analysis showed that perceived similarity had a significant effect on mass trust. The findings are consistent with those of Dwyer et al. (1987), Morgan and Hunt (1997), and McKnight et al. (1998). It is speculated that the reason may be that perceived similarity tends to produce cognitive consistency, and individuals usually believe in other individuals who are similar to themselves. A study by Fu et al. (2018) showed that interpersonal similarity has a positive effect on interpersonal trust. According to interpersonal attraction theory, perceived similarity triggers interpersonal attraction, i.e., individuals are easily attracted to people who are similar to themselves. Interpersonal attraction affects individuals' perceptions of others; individuals usually have higher identification with those who are attracted to them and tend to make positive evaluations. In addition, interpersonal attraction affects individuals' behavior, as individuals tend to interact with those who are attracted to them and expect to increase the frequency and depth of their interactions.

In the context of short video applications, since the public and individuals have similar characteristics, the public can infer the traits of individuals based on their own knowledge, thus reducing uncertainty about individuals and increasing mass trust. When people interact with similar individuals, they can achieve better communication and understanding, reduce disagreements and conflicts, and enhance their intimacy, thus increasing their trust in them. Therefore, as the degree of similarity between individuals and others increases, individuals' trust in the user will gradually increase.

Fourth, the results of data analysis verified the mediating role of perceived similarity on the relationship between self-disclosure and mass trust. The findings are consistent with the inferences of Hitsch et al. (2010), Liu et al. (2017), Zhan and Du (2018), and Yan and Fu (2021). In the context of short video applications, interactions between individuals affect the generation of perceived similarity, which in turn stimulates intimacy such as interpersonal trust and thus a series of attraction effects. In the short video application context, if individuals publicly disclose richer information, then individuals can rely on self-disclosure of public information to inspire others to produce perceived similarity and use it as a means of attracting others and determining perceptions and behaviors toward others. In the context of individual self-disclosure, individuals can infer from self-disclosure that they are similar to similar individuals, generate interpersonal attraction, hold high identification and positive evaluation of individuals, and thus form trust in individuals. Related studies have shown that good interaction between users in short video application contexts positively affects perceived similarity, which in turn stimulates interpersonal attraction and raises relationship levels. It can be inferred that the more adequate information about an individual's self-disclosure, the stronger similarity the individual perceives and the higher the level of trust in the individual.

Fifth, the results of data analysis showed that trust disposition has a moderating effect on the relationship between perceived similarity and mass trust. The findings are consistent with the inferences of Daft et al. (1987), Shen et al. (2010), Cheng et al. (2016), and Fu et al. (2018). Trust disposition reflects the characteristics that individuals have in themselves, not the trust intention or attitude of individuals toward a specific object. This individual characteristic is mainly related to the individual's cultural background, life environment and past experiences, so there will be large or significant differences in the trust disposition of different individuals. Trust disposition reflects individual disposition, and when it is combined with individual perceived similarity in perceiving the trustworthiness of others, it can promote positive influence of individual perceived similarity on mass trust and work together to strengthen the trust formed in mass, so trust disposition as a moderating variable can influence the relationship between individual perceived similarity and mass trust. In the short video application context, when individuals' trust disposition is low, individuals are less likely to trust others, so even if individuals have more perceived similarity, it is difficult for them to have a higher degree of trust in individuals. On the contrary, when individuals' trust disposition is high, individuals are more likely to trust others, and the more individuals perceived similarity, the higher their trust will be. It can be seen that individual trust disposition promotes the positive effect of perceived similarity on mass trust, i.e., the higher the individual trust disposition, the greater the positive effect of perceived similarity on mass trust.

### Theoretical contributions

First, empirical studies on the factors influencing mass trust are relatively limited, except for the studies by Wang and Stefanone (2013) and Li et al. (2018), Previous studies have mainly explored the motives for the formation of public trust and its mechanisms of action from a qualitative perspective. Therefore, the research results lack the support of empirical tests, and the accuracy of the research findings is questioned. This study analyzes the data collected from the questionnaire survey by means of structural equation modeling. Therefore, this study fills the gap in the research on the factors influencing public trust to some extent and enriches the results of empirical research in the field of public trust.

Second, this research discusses the mechanisms by which self-disclosure affects mass trust based on social exchange theory. According to the specificity of short video usage context, the mediating role of perceived similarity and the moderating role of trust disposition are introduced into the empirical research model of the relationship between self-disclosure and mass trust, which extends the extension of the theoretical framework and enhances the generalizability of the study findings. Therefore, this study reveals and enriches the research perspective and content of self-disclosure and mass trust by exploring the relationship between “how self-disclosure affects mass trust”.

Third, this study further tested that the effect of individual self-disclosure on mass trust increased as the level of trust disposition increased, i.e., trust disposition had a moderating role in the relationship between self-disclosure predicting mass trust. Therefore, this article explores the impact of self-disclosure on public trust through structural equation modeling, which fills the gap of previous studies on the impact of public trust in terms of empirical research, enriches the mediating mechanism of explaining public trust from the perspective of self-disclosure, and provides a reference for future related research.

### Practical implications

First, enhancing self-disclosure is an important driver of motivating mass trust. This study shows that individual's self-disclosure has a significant positive effect on mass trust. Therefore, short-video applications should pay attention to the relevance of formulating constrained individual disclosures, and play a catalytic role in stimulating individual self-disclosure. In addition, through policy guidance, users are made aware of the importance of self-disclosure in the context of short video applications. Self-disclosure not only reduces mass' perceived uncertainty, but also creates a sense of familiarity and intimacy among individuals, thus enhancing intimacy. In terms of specific measures, individuals can present their similarity to the target group in terms of values, interests and attitudes on short video applications, thus enhancing mass trust.

Second, establishing high-quality perceived similarity is an effective strategy to indirectly enhance mass trust. The results of this study suggest that self-disclosure can have an indirect positive impact on mass trust through the mediating role of perceived similarity. Therefore, individuals can alleviate self-stress, loneliness and isolation through the disclosure of quality information on short video applications, drive others to generate identification with self, get warmth, get help and love from it, and thus eliminate or reduce the sense of stress, loneliness and isolation, i.e., gain perceived similarity. Therefore, individuals get to know more friends through short video applications, maintain communication and contact, gain friendship, support and respect, and thus gain a sense of belonging such as identity, which in turn further enhances the trust formed between individuals on the basis of perceived similarity.

Third, segmentation of target markets is an effective way to classify and meet the differential needs of different groups. This study finds that trust disposition positively moderates the relationship between perceived similarity and mass trust. This indicates that the higher the level of trust disposition is, the stronger the effect of perceived similarity on mass trust. In this regard, short video applications should focus their investments on gathering such users, cultivating and maintaining users with higher levels of trust disposition, thus significantly influencing the role of perceived similarity driving mass trust. However, for users with low levels of trust disposition, the impact of perceived similarity on mass trust may be more moderate as the level of trust disposition changes. For such users, short video applications can focus on the traditional marketing strategy level, i.e., increase investment in the construction of self-disclosure and mass trust relationships. For example, creating better intimacy for users by optimizing the quality of information disclosure.

### Research limitations and future research directions

First, this study suffers from data statics and cross-sectional limitations. The data collected in this study provided initial support for the hypothesis, but because the key variables were self-reported at the same time, it resulted in data analysis results that were subject to common method bias. Although statistically validated to have a small effect, this may still increase the error in the data analysis (Podsakoff et al., 2012). In addition, although hypothesis testing has well established a positive relationship between self-disclosure and mass trust, it is still not possible to infer the direction of the relationship between self-disclosure and mass trust from cross-sectional data. Therefore, in future studies, these limitations can be addressed using a two-wave online panel sample and a dynamic tracking method to examine the trend of mass trust to enhance the persuasiveness and rigor of the study findings.

Second, although this study uses TikTok as a sample to collect data and the findings are significantly representative, the sample scope of this study is from China and the theoretical nature of the findings needs to be further validated by different short video applications in a larger context. Therefore, future research can conduct data collection in a broader scope, such as Twitter, Facebook, KakaoTalk and other foreign short video applications with obvious social features. A larger sample group can be used for group comparison analysis, even for short video applications in foreign developed countries; individual differences in different cultures and regional and national contexts can be explored to provide marketing strategies for short video applications in different market segments, thus enhancing the generalizability of the research results.

## Data availability statement

The raw data supporting the conclusions of this article will be made available by the authors, without undue reservation.

## Ethics statement

Ethical review and approval were not required for the study on human participants in accordance with the local legislation and institutional requirements. Informed consent was obtained from all subjects involved in the study.

## Author contributions

Conceptualization and methodology: AR, OS, and DJ. Formal analysis and investigation: YZ. Writing original draft and writing-review and editing, AR, OS, DJ, and YZ. All authors have read and agreed to the published version of the article.

## Conflict of interest

The authors declare that the research was conducted in the absence of any commercial or financial relationships that could be construed as a potential conflict of interest.

## Publisher's note

All claims expressed in this article are solely those of the authors and do not necessarily represent those of their affiliated organizations, or those of the publisher, the editors and the reviewers. Any product that may be evaluated in this article, or claim that may be made by its manufacturer, is not guaranteed or endorsed by the publisher.
